# Effect of Phosphoric Acid on the Preparation of α-Hemihydrate Gypsum Using Hydrothermal Method

**DOI:** 10.3390/ma16175878

**Published:** 2023-08-28

**Authors:** Jianwu Zhang, Xiao Wang, Pengtao Hou, Biao Jin, Xiaoting Zhang, Zhixin Li

**Affiliations:** School of Materials and Chemical Engineering, Henan University of Urban Construction, Pingdingshan 467036, China; 30010906@huuc.edu.cn (X.W.); pengtao2103220019@163.com (P.H.); 20131001@huuc.edu.cn (B.J.); 30010910@huuc.edu.cn (X.Z.); 20171001@huuc.edu.cn (Z.L.)

**Keywords:** hydrothermal method, phosphoric acid, α-hemihydrate gypsum, crystalline transformation, regulation

## Abstract

The effects of phosphoric acid (H_3_PO_4_) in pressurized aqueous solution on the dehydration of CaSO_4_·2H_2_O to form α-hemihydrate gypsum (α-HH) phase and the regulation of crystal shape were studied in this paper in order to provide guidance for the low-cost and high-value utilization of phosphogypsum. The results showed that H_3_PO_4_ can significantly accelerate the formation rate of the α-HH phase and that it did not participate in the formation of the α-HH phase in the form of eutectic phosphorus during crystalline phase transformation. In terms of crystal shape regulation, H_3_PO_4_ can impact the effect of a citric acid crystal regulator on α-HH crystal shape regulation. The more H_3_PO_4_ added, the greater the aspect ratio of α-HH. Accordingly, the water consumption and 2 h dry compressive strength of α-HH products were gradually increased and decreased with an increase in H_3_PO_4_ content, respectively. Despite this, the compressive strength of α-HH can still meet the requirements of the α20 grade in JC/T 2038-2010 “α High Strength Gypsum” in China when the H_3_PO_4_ content was limited to less than 0.4%.

## 1. Introduction

Phosphogypsum is a by-product of the wet process phosphoric acid industry [1,2,3]. Approximately 5 tons of phosphogypsum are generated per ton of phosphoric acid production [4]. At present, the annual emission of phosphogypsum in the world has exceeded 280 million tons, but the comprehensive utilization rate is only about 10% [5,6]. In recent years, China, as a big country with phosphate mineral resources, produces and dissolves about 70 million tons of phosphogypsum every year, and the utilization rate is only 40% [2]. A large amount of unused phosphogypsum is mainly deposited, which not only occupies a large amount of land resources but also causes serious environmental pollution problems [7,8,9]. Therefore, it is urgent to accelerate the efficient utilization of phosphogypsum.

The main chemical composition of phosphogypsum is calcium sulfate dehydrate (CaSO_4_·2H_2_O), making up over 90% of it. Therefore, phosphogypsum can be used to effectively replace natural gypsum to produce α-hemihydrate gypsum (α-HH). This is one of the key ways to realize the high-value utilization of phosphogypsum resources and also one of the most promising and beneficial treatment methods [10,11,12,13]. Moreover, due to the powdery nature of phosphogypsum, it is very suitable to transform it into α-HH by using a hydrothermal method. [12,14,15]. However, unlike natural gypsum, phosphogypsum contains a large number of harmful impurities, such as soluble phosphorus (P_2_O_5_), soluble fluorine (F^−^), insoluble matter, organic matter, etc. [16,17,18,19]. Many studies have shown that whether phosphogypsum is used to prepare α-HH, producing gypsum and gypsum products results in various harmful impurities in the phosphogypsum, which have a significant negative impact on its application performance in most cases. However, since the mechanisms of influence on impurities in phosphogypsum are not fully understood, they must be eliminated. In order to avoid the negative impact of the impurity in phosphogypsum, a series of methods can be implemented, such as water washing, lime neutralization, calcination, and other methods to pretreat phosphogypsum before using it. These conventional techniques greatly increase the cost of using phosphorus gypsum and are not conducive to the resource utilization of phosphogypsum [20,21,22]. Therefore, it is of great theoretical value and practical significance to deeply explore the influence of impurities in phosphogypsum on its application performance and put forward control criteria in terms of impurities to realize low-cost and efficient utilization of phosphogypsum resources.

Many studies have shown that soluble P_2_O_5_ is the most harmful among the various impurities contained in phosphogypsum. The soluble P_2_O_5_ impurities in phosphogypsum mainly come from residual phosphoric acid (H_3_PO_4_), which mainly exists in the form of H_3_PO_4_, H_2_PO_4_^−^ and HPO_4_^2−^ [23,24]. The greatest harm of soluble P_2_O_5_ impurities is that it can cause the pH of the reaction liquid system to change when phosphogypsum is used to prepare α-HH, which will inevitably affect the crystallization habit of α-HH and ultimately affect the physical and mechanical properties of the product [25,26]. To avoid the harm of impurities, most current practices include the pre-purification of phosphogypsum before it is used in the production of α- HH, which significantly increases the utilization cost of phosphogypsum and is not conducive to its high-value utilization. Therefore, it is very important to explore the influence law of harmful impurities in phosphogypsum in the α-HH production process and reveal the influence mechanism of impurities, which is the key to realizing green, low-cost, and high-value utilization of phosphogypsum resources. Thus, this study synthesized α-HH by using a hydrothermal method and studied the influence of H_3_PO_4_ (P_2_O_5_) on the crystal phase transformation of calcium sulfate, the morphology characteristics of α-HH, and the physical–mechanical properties of α-HH during this process. On this basis, the influence mechanisms of H_3_PO_4_ on the preparation and performance of hemihydrate gypsum were revealed. The research in this paper can provide guidance for green, low-cost, and high-value utilization of phosphogypsum resources.

## 2. Materials and Methods

### 2.1. Materials

**Figure 1:**
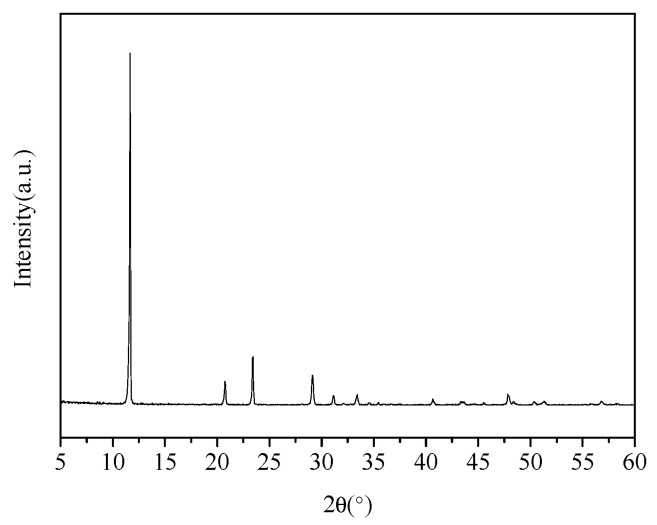
XRD spectrum of pure gypsum for the experiment.

**Figure 2:**
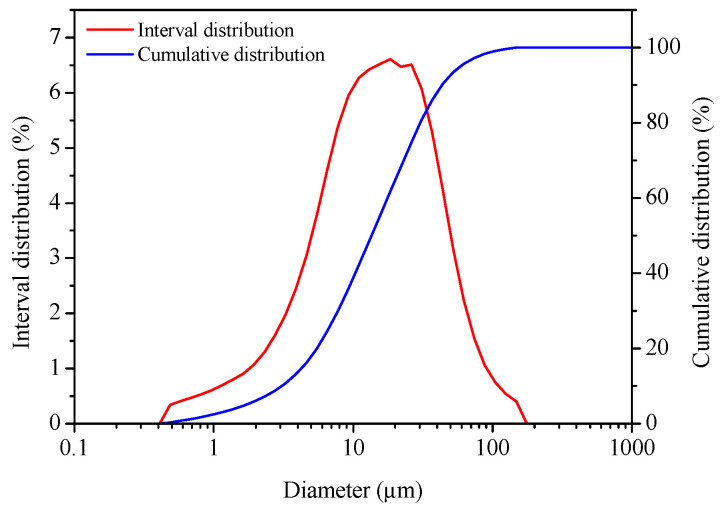
Particle size distribution of pure gypsum for the experiment.

The pure gypsum used in the experiment was taken from Tianjin kemio Chemical Reagent Co., Ltd. (Tianjin, China), where the content of CaSO_4_·2H_2_O was ≥99%. The XRD spectrum and particle size distribution of pure gypsum are shown in Figure 1 and Figure 2, respectively. Phosphoric acid was bought from Yantai Shuangshuang Chemical Co., Ltd. (Yantai, China), which had a H_3_PO_4_ concentration of 85 wt.%. Citric acid was used as a crystal modifier, purchased from Tianjin Bodi Chemical Co., Ltd. (Tianjin, China). Anhydrous ethanol was used to wash the synthesized α-hemihydrate gypsum (α-HH) samples and immobilize the crystal form, purchased from Yansheng Chemical Co., Ltd. (Tianjin, China). The deionized water was prepared using a lab tower EDI pure water instrument.

### 2.2. Preparation of α-HH

A hydrothermal reactor (500 mL) was adopted to prepare α-HH products. The specific procedures were as follows:

Firstly, 120 g of pure gypsum was mixed, and 280 g of deionized water was accurately weighed and prepared in the reaction slurry with a slurry concentration of (ca.) 30%. Secondly, H_3_PO_4_ and citric acid were measured and mixed with the reaction slurry according to the experimental requirements. Afterward, the prepared slurry was placed in the reactor and sealed, and α-HH was prepared at 130 °C and under 260 r/min stirring conditions. The solid material in the reactor was fully filtered and washed with absolute ethanol immediately after reaching the set reaction time. Finally, the obtained samples were dried at 50 °C for 5 h in an oven to obtain the final α-HH product.

### 2.3. Experimental Methods

#### 2.3.1. XRD Test

The prepared α-HH samples were analyzed using X, Pert Pro X-ray diffractometer (XRD), produced by Malvern Panalytical in the Netherlands. A Cu Ka emission source was used for testing. The test range was 5–60° at a scanning rate of 10 °/min. This test can clarify the crystal transformation of gypsum in the prepared sample.

#### 2.3.2. Morphology Test

The morphology of the α-HH samples was observed using a CI-L material microscope produced by Nikon. The specific steps were as follows:

Firstly, a small number of samples were taken on the slide, and the samples were dispersed with absolute ethanol. After drying, the samples were placed on the sample table of the material microscope. The magnification of the eyepiece was adjusted to 400× to observe the morphological characteristics of the prepared α-HH, and the diameter and length of the crystal were measured and analyzed.

#### 2.3.3. Infrared Spectrum Test

The chemical groups of prepared α-HH samples were characterized using a Fourier transform infrared spectrophotometer (FTIR; Nicolet iS10; Thermo Fisher Scientific Corporation, Waltham, MA, USA) with a scanning range from 500 cm^−1^ to 4000 cm^−1^. The aim of this test was to observe whether H_3_PO_4_ was involved in the formation of α-HH in the form of eutectic phosphorus.

#### 2.3.4. Determination of Water of Crystal Content

In order to evaluate the effect of H_3_PO_4_ on α-HH conversion, the content of crystalline water in the samples was analyzed. The specific test process was as follows: 

A certain number of prepared samples were calcined in an oven at 230 ± 5 °C to a constant weight. According to the change of sample mass before and after calcination, the content of water and crystal in the sample was calculated with Formula (1).

(1) $$W=\frac{m_{0}-m_{1}}{m_{0}}\times 100\%$$

where:

W—Water of crystallization content, %;
$m_{0}$—Mass of sample before calcination, g;
$m_{1}$—Mass of sample after calcination, g.

#### 2.3.5. Physical and Mechanical Properties Test

The water consumption of the standard consistency of the prepared α-HH samples was tested according to Gypsum—Determination of physical properties of pure paste (GB/T 17669.4-1999) [27]. The samples were transformed into uniform slurry and molded into test blocks of 40 mm × 40 mm × 40 mm under the water consumption of standard consistency. After 30 min, the castings were demoulded and then cured for 2 h under natural conditions. The prisms were dried to a constant weight at 60 °C and readied for the mechanical properties test. The mechanical strength was tested using an automated breaking and compression resistance tester (WAY-300, Xiyi Building Material Instrument Factory, Wuxi, China).

## 3. Results and Discussion

### 3.1. X-ray Diffraction Analysis

The formation of α-HH was a process of dissolution and recrystallization. Under the condition of pressurized water vapor, the dihydrous gypsum in phosphogypsum first removed two-thirds of crystalline water to form a α-HH crystal embryo. This was in the environment of liquid water and quickly dissolved under suitable temperature conditions, making the concentration of α-HH in the liquid phase increase continuously. When it reached saturation, the α-HH rapidly crystallized and formed. However, the acidification effect and impurity effect caused by the raw material characteristics of phosphogypsum were bound to have a certain degree of influence on the thermodynamics and kinetics of the reaction process. This, in turn, would affect the crystal phase transition process, which was obviously directly related to the reaction efficiency and would thus alter the process cost. Therefore, combined with the starting point of this study, the effect of H_3_PO_4_ on the α-HH crystal phase transformation process without a crystal regulator was initially studied.

**Figure 3:**
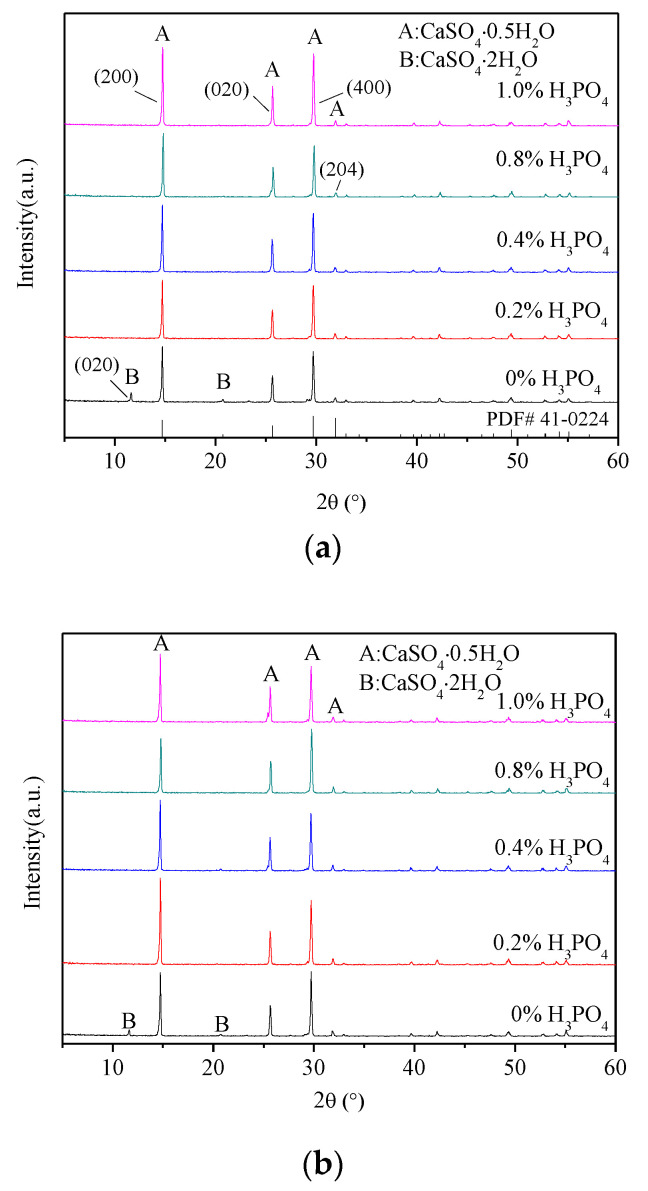

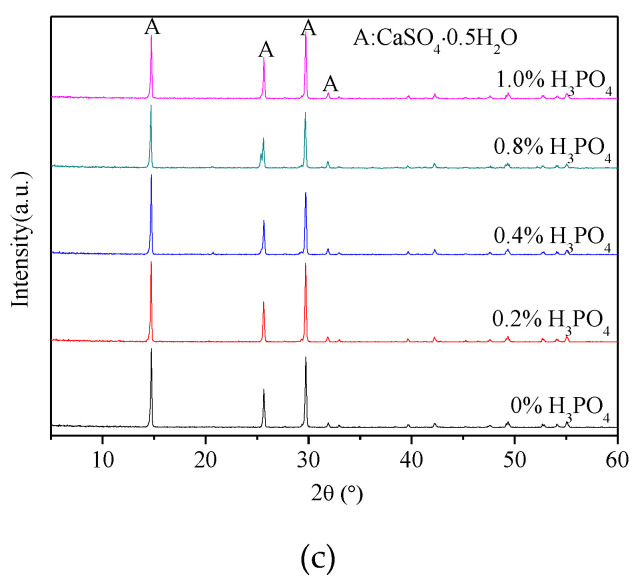
XRD patterns of each sample under different H_3_PO_4_ incorporations and reaction durations ((**a**): 0.5 h; (**b**): 1 h; (**c**): 1.5 h).

Figure 3 shows the XRD patterns of the samples prepared under the conditions of 0.5 h, 1.0 h and 1.5 h reaction times of the reaction system with different H_3_PO_4_ contents. For the reaction system without H_3_PO_4_, a considerable amount of α-HH was detected after only 0.5 h of reaction time, but there was still a certain amount of dihydrate gypsum that had not been completely transformed. The characteristic peak of dihydrate gypsum disappeared until the sample was reacted for an extra 1.5 h, indicating that it had completely changed to α-HH at this time. The transition process of the crystal phase can be entirely completed after 0.5 h of reaction time when 0.2% H_3_PO_4_ was added, which revealed that the existence of H_3_PO_4_ can significantly improve reaction efficiency. With the increase in H_3_PO_4_ content, the time of complete conversion of dihydrate gypsum to α-HH remained within 0.5 h without significant change. The main reason why H_3_PO_4_ can accelerate the formation rate of α-HH is because H_3_PO_4_ can reduce the pH value of the reaction system. This, in turn, increases the supersaturation of α-HH and reduces the surface free energy at the interface between crystal and reaction liquid, which was conducive to the rapid formation of the α-HH phase [25]. At the same time, further analysis of Figure 3, especially Figure 3a, clearly shows that the incorporation of phosphoric acid can also affect the crystallinity of α-HH. The (400) crystal plane of α-HH was parallel to the crystal *C*-axis, while (204) was a crystal plane perpendicular to its *C*-axis. With the addition of phosphoric acid, the ratio of (400) crystal plane diffraction peak intensity to (204) crystal plane diffraction peak intensity basically increased. This showed that phosphoric acid can affect the growth rate of different crystal faces, promote the growth along the *C*-axis direction, and inhibit the growth in the diameter direction so that the crystal morphology was highly fine-needle-like. The results were also consistent with those of crystal morphology analysis.

### 3.2. Crystal Water Content

**Figure 4:**
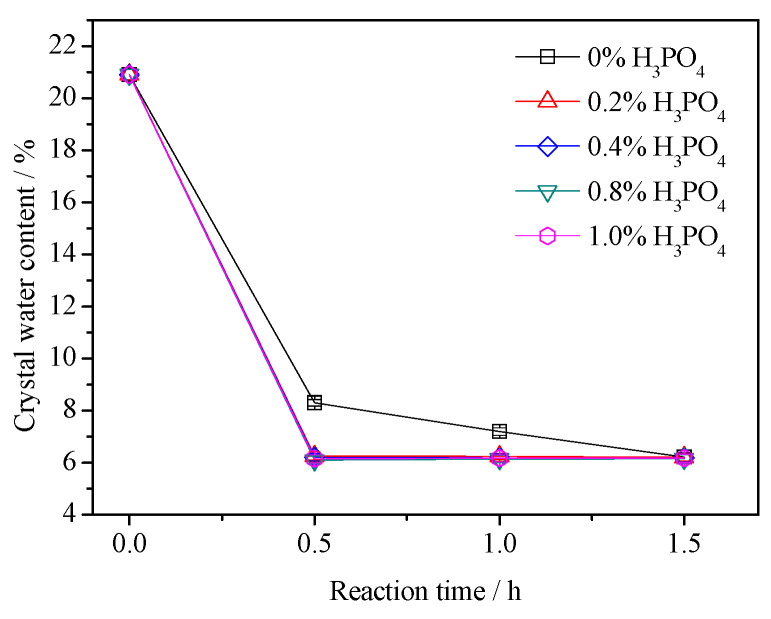
The test results of crystal water content.

The theoretical crystalline water content of dihydrate gypsum and α-HH was 20.9% and 6.21%, respectively. Therefore, the formation of α-HH can be reflected by measuring the content of crystal water in the samples. The crystal water content of each sample was measured, which is shown in Figure 4. For the reaction system without H_3_PO_4_, the crystalline water content of the prepared sample decreased significantly from 20.9% to 8.3% after a 0.5 h reaction time. This indicated that a considerable amount of α-HH had been formed at this time. After 1.5 h, the water of crystallization content was 6.22%, which was very close to the theoretical water of crystallization content of α-HH, revealing that the dihydrate gypsum crystal had all transformed into the α-HH phase at this time. For each reaction system with different amounts of H_3_PO_4_, the crystal water content of the obtained products was close to the theoretical crystal water content of α-HH after a 0.5 h reaction time, which indicated the crystal transformation process had been fully completed at this time. In general, the results of the crystal water content were consistent with the XRD result. This further supports the experimental conclusion that the presence of H_3_PO_4_ can promote the rapid formation of the α-HH phase.

### 3.3. Infrared Analysis

**Figure 5:**
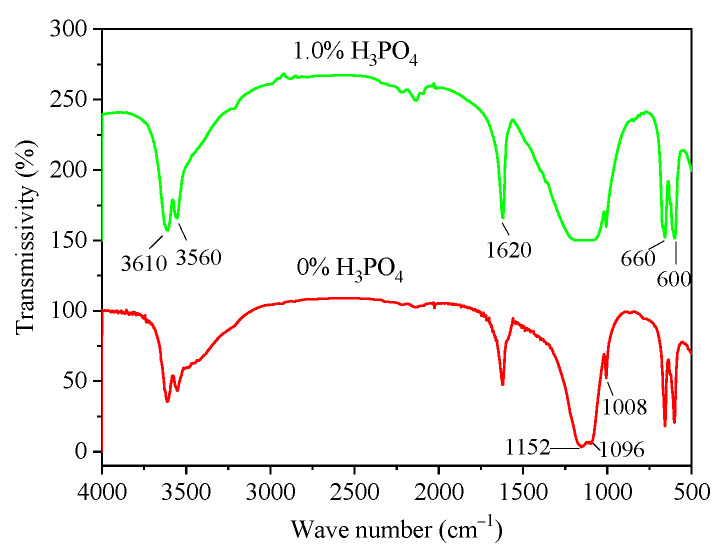
Infrared spectrum test results.

Due to self-ionization, H_3_PO_4_ can exist in the form of H_3_PO_4_, H_2_PO_4_^−^ and HPO_4_^2−^ in the reaction liquid phase and can combine with Ca^2+^ ions in the liquid phase to form various forms of calcium phosphate. Calcium phosphate salts, especially those containing HPO_4_^2−^, had similar lattice constants with calcium sulfate salts. Therefore, it was easy to place SO_4_^2−^ into the α-HH lattice to form a eutectic phosphorus solid solution. Once eutectic phosphorus formed, the hydration and hardening properties of the prepared α-HH can be adversely affected. In order to reveal whether eutectic phosphorus can be produced in the process of α-HH preparation, two kinds of α-HH samples prepared with H_3_PO_4_ contents of 0% and 1.0% were analyzed via infrared spectroscopy. The results are shown in Figure 5. The infrared characteristic absorption peak of eutectic phosphorus was usually located near 840 cm^−1^, and no characteristic peak of eutectic phosphorus was found in the two groups of samples via a comparative test [28]. At the same time, the characteristic peaks of all kinds of calcium phosphate salts were not detected, which may be due to the low amount of phosphate acid incorporation, resulting in the low content of calcium phosphate salts.

### 3.4. Morphology Analysis

**Figure 6:**
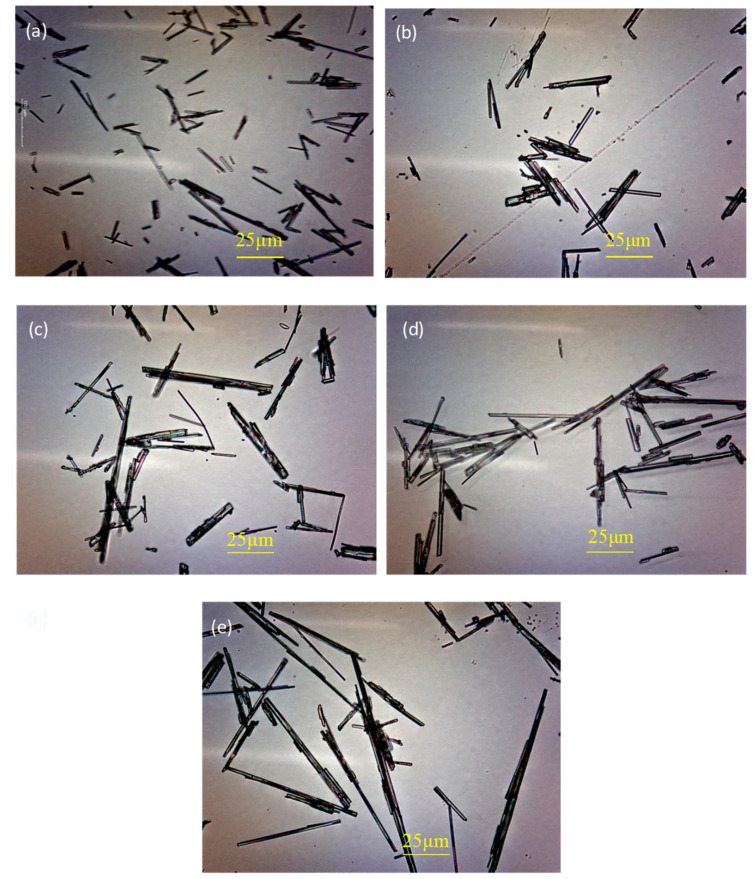
Morphology of α-HH with different H_3_PO_4_ content ((**a**): 0% H_3_PO_4_; (**b**): 0.2% H_3_PO_4_; (**c**): 0.4% H_3_PO_4_; (**d**): 0.8%H_3_PO_4_; (**e**): 1.0% H_3_PO_4_).

In order to analyze the effect of H_3_PO_4_ on the morphology of α-HH, the morphology of each sample prepared under the 1.5 h reaction time was observed, as shown in Figure 6. Without H_3_PO_4_, α-HH presented a fine-needle-like crystalline form with a diameter of 2–5 μm, length of 15–80 μm and aspect ratio of 4–16. The addition of H_3_PO_4_ did not change the crystal morphology of α-HH but caused a significant increase in the aspect ratio of the α-HH crystal. And with the increase in H_3_PO_4_ content, the aspect ratio of the crystal also gradually increased. When H_3_PO_4_ content was as high as 1.0%, the aspect ratio of α-HH increased significantly to 44–60. The main reason for this phenomenon was that H^+^ in the reaction solution can combine with SO_4_^2−^ to form HSO_4_^−^ with the addition of H_3_PO_4_, which may increase the free SO_4_^2−^ concentration in the solution. The growth rate of the (111) crystal plane located at the top of the α-HH crystal can be accelerated. Accordingly, the aspect ratio of the crystal increased.

**Figure 7:**
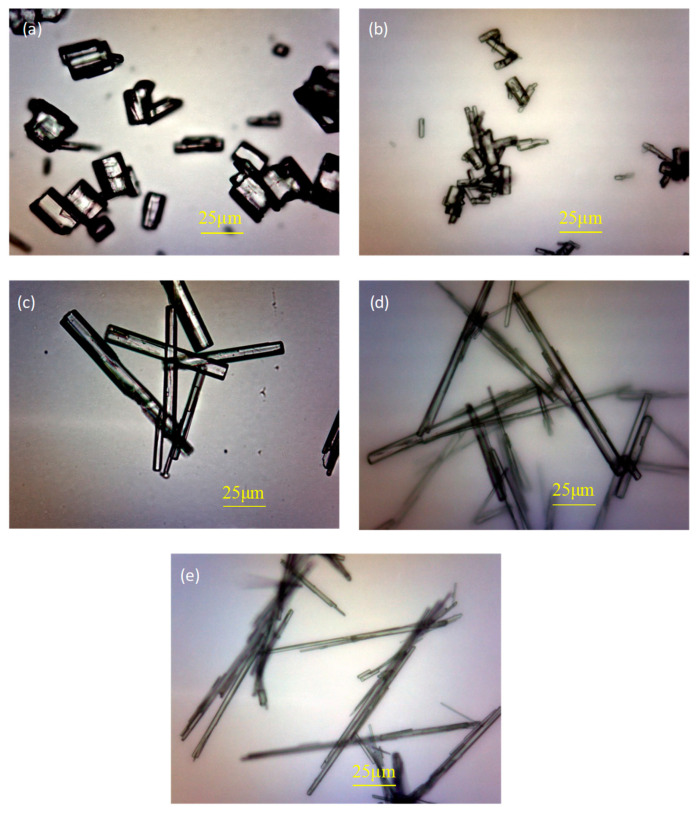
Effect of phosphoric acid on the morphology of samples after citric acid crystallization ((**a**): Citric acid + 0% H_3_PO_4_; (**b**): Citric acid + 0.2% H_3_PO_4_; (**c**): Citric acid + 0.4% H_3_PO_4_; (**d**): Citric acid + 0.8% H_3_PO_4_; (**e**): Citric acid + 1.0% H_3_PO_4_).

Many studies have shown that it is necessary to add some crystal modulators to regulate the crystal morphology of α-HH in the process of preparation in order to give good physical and mechanical properties. The performance was best when the aspect ratio of the crystal was close to 1:1. Therefore, an appropriate amount of citric acid was introduced into the reaction system, and the morphology of each α-HH sample prepared is shown in Figure 7. Figure 7a shows the morphology of α-HH prepared by the reaction system without H_3_PO_4_ doped and regulated by citric acid. It can be seen by comparison with Figure 7a that the morphology of the α-HH crystal changed from the original fine-needle-like shape to a short column shape after the addition of citric acid. The aspect ratio of the crystal also decreased to 1:1, which indicated citric acid had an excellent crystal-regulating effect. It can be seen from Figure 7b–e that H_3_PO_4_ had a negative impact on the crystal-regulating effect of citric acid, and the larger the amount of H_3_PO_4_, the more significant the effect. When 0.2% H_3_PO_4_ was added, the aspect ratio of α-HH only increased to 2:1. When the content increased to 0.4%, α-HH still showed columnar morphology on the whole, and the aspect ratio only increased to around 6:1. However, the morphology of α-HH showed an obvious fine-needle-like shape when the H_3_PO_4_ content was more than 0.8%, and the crystal aspect ratio increased to more than 20:1.

The main reason why phosphoric acid can impact the crystal-regulating effect of citric acid was that H_3_PO_4,_ as a kind of medium–strong acid, can change the pH of the liquid reaction system, and then cause the ionization process of citric acid molecules. In fact, there would be a three-order dissociation equilibrium in the reaction solution when citric acid (abbreviated as H_3_Cit), which is categorized as a ternary weak acid, was added to the reaction system. In the first stage, H_3_Cit was ionized to H_2_Cit^−^ and H^+^. In the second stage, H_2_Cit^−^ was further ionized to HCit^2−^ and H^+^. Finally, HCit^2−^ was further ionized to Cit^3−^ and H^+^. The complexation of H_2_Cit^−^, HCit^2−^ and Cit^3−^ carboxylate ions was generated due to the ionization of citric acid in the reaction liquid phase with Ca^2+^ on the crystal surface of the α-HH crystal (111). This was mainly due to the fact that calcium ions located on the (111) crystal plane of α-HH can be complexed with H_2_Cit^−^, HCit^2−^ and Cit^3−^ complex anions. These were generated due to the ionization of citric acid, which significantly decreased the growth rate of the crystal along the *C*-axis and made α-HH develop in the short columnar direction [29]. Therefore, when H_3_PO_4_ was not added to the reaction system, citric acid could easily ionize and produce more complex anions than in a neutral environment, which significantly regulated the crystal morphology of α-HH. The addition of H_3_PO_4_ can significantly increase the acidity of the reaction liquid phase and then affect the degree of dissociation of citric acid molecules. Moreover, the greater the amount of H_3_PO_4_, the more significant this effect can become, which leads to a greatly reduced crystal-regulating effect of citric acid.

### 3.5. Water Consumption for Standard Consistency

**Figure 8:**
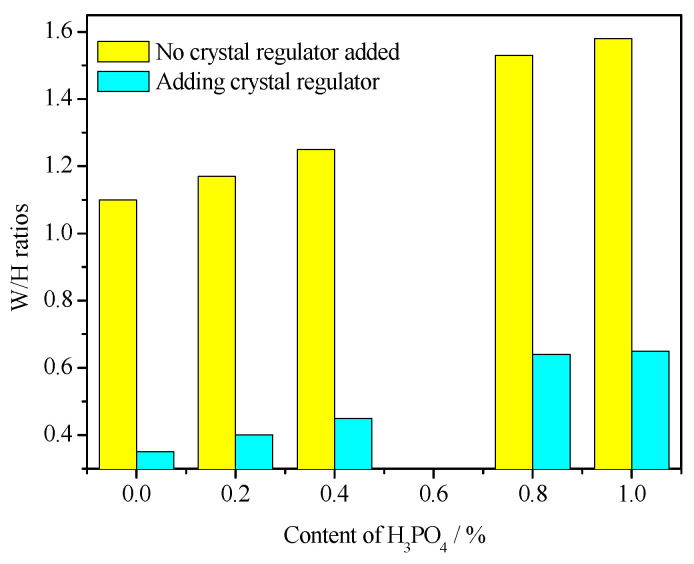
Effect of H_3_PO_4_ on the water consumption for standard consistency of α-HH.

Figure 8 shows the influence of H_3_PO_4_ on the water consumption for standard consistency of α-HH with and without a citric acid crystal regulator. Without a citric acid crystal regulator, the α-HH crystal was presented in a fine-needle-like shape (Figure 6), and the aspect ratio of the crystal was significantly larger. Accordingly, the water consumption of its standard consistency was considerably greater. The addition of H_3_PO_4_ further increased the aspect ratio of α-HH. Therefore, the water consumption of standard consistency of α-HH also gradually increased with the increase in H_3_PO_4_ content. After citric acid was added, the water consumption of α-HH at standard consistency decreased significantly under the regulation of its crystal form. For example, for samples without phosphoric acid, the water consumption for standard consistency decreased significantly from 1.1 to 0.35 before and after citric acid incorporation. However, due to how H_3_PO_4_ can significantly impact the regulatory effect of citric acid, the water consumption of α-HH standard consistency increased significantly with H_3_PO_4_ incorporation, and the higher the H_3_PO_4_ content, the higher the water consumption. However, with the same amount of H_3_PO_4_, the water requirement of α-HH prepared with a citric acid crystal regulator was significantly lower than that without citric acid.

### 3.6. Mechanical Properties

**Figure 9:**
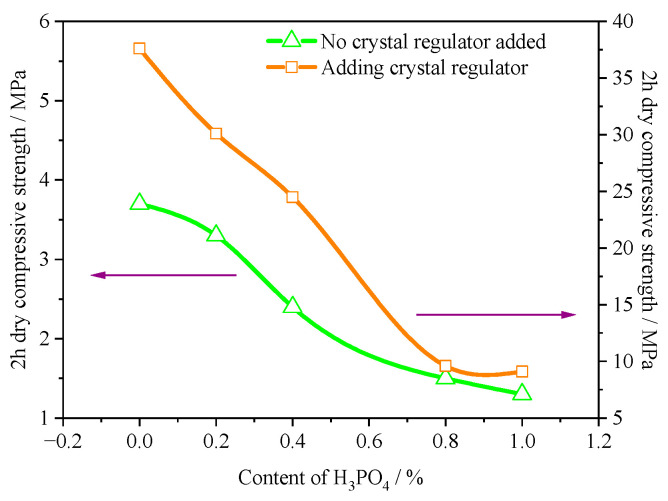
Effect of H_3_PO_4_ on 2 h dry compressive strength of α-HH.

The test results of mechanical properties are shown in Figure 9. When the citric acid crystal regulator was not added, the α-HH samples had a fine-needle-like shape, which led to higher water consumption of standard consistency, resulting in a significantly lower strength. With the increase in H_3_PO_4_ content, the strength can be further reduced. After the crystal regulation of citric acid, the strength of α-HH was significantly increased due to the significant reduction in water consumption for standard consistency of α-HH. For example, the strength of α-HH samples with 0% H_3_PO_4_ content increased significantly from 3.7 MPa to 37.6 MPa before the inclusion of citric acid. According to the Chinese standard (JC/T 2038-2010 “α High Strength Gypsum”) [30], it can meet the requirements of α30 strength grade. Similarly, due to the negative effect of H_3_PO_4_, the strength of α-HH decreased obviously with the incorporation of H_3_PO_4_. Moreover, the higher the H_3_PO_4_ content, the more the strength decreased. However, the strength of α-HH was still high, up to 24.5 MPa when the content of H_3_PO_4_ was 0.4%, which meets the requirements of α20 strength grade.

## 4. Conclusions

This paper mainly studied the effect of H_3_PO_4_ on the crystal transformation, crystal regulation and physical mechanical properties of α-HH in the process of hydrothermal synthesis and aimed to provide necessary guidance for the green, low-cost, and high-value utilization of phosphogypsum. The important conclusions are as follows:

(1) The presence of H_3_PO_4_ can accelerate the formation rate of the α-HH phase. The transition time of all gypsum to the α-HH phase can be shortened from 1.5 h to 0.5 h when only 0.2% H_3_PO_4_ was added.
(2) The infrared test results showed that H_3_PO_4_ did not enter the α-HH lattice to form eutectic phosphorus solid solution. In addition, the presence of related calcium phosphate was not detected in α-HH, possibly due to the small amount of phosphate content leading to the small amount of calcium phosphate production.
(3) The addition of H_3_PO_4_ can significantly affect the crystal shape of α-HH and significantly weaken the regulatory effect of citric acid on the crystal shape of α-HH, resulting in a significant increase in the aspect ratio of α-HH crystals. Moreover, the higher the H_3_PO_4_ content, the more significant the negative impact.
(4) Due to the significant increase in the aspect ratio of α-HH caused by H_3_PO_4_, the standard consistency water consumption of α-HH gradually increased with the increase in H_3_PO_4_ content. Accordingly, the strength of the hardened body of α-HH gradually decreased. However, the prepared α-HH can still meet the requirements of α20 high-strength gypsum when the H_3_PO_4_ content was less than 0.4%.
(5) It can be inferred from this study that the soluble P_2_O_5_ impurities present in phosphogypsum can effectively promote the rapid formation of the α-HH phase when phosphogypsum is used for hydrothermal preparation of high-strength gypsum, but it can have a significant negative effect on the crystal regulation of α-HH. Therefore, the screening of suitable crystallization agents to avoid the adverse effects of soluble P_2_O_5_ will be one of the focuses of future research in this field.

## Data Availability

Not applicable.
